# Microarray Analysis Reveals Distinct Gene Expression Profiles Among Different Tumor Histology, Stage and Disease Outcomes in Endometrial Adenocarcinoma

**DOI:** 10.1371/journal.pone.0015415

**Published:** 2010-11-08

**Authors:** Paulette Mhawech-Fauceglia, Dan Wang, Joshua Kesterson, Kimberly Clark, Laketa Monhollen, Kunle Odunsi, Shashikant Lele, Song Liu

**Affiliations:** 1 Department of Pathology, Roswell Park Cancer Institute, Buffalo, New York, United States of America; 2 Department of Biostatistics, Roswell Park Cancer Institute, Buffalo, New York, United States of America; 3 Department of Gynecology-Oncology Surgery, Roswell Park Cancer Institute, Buffalo, New York, United States of America; 4 Department of Cancer Genetics, Roswell Park Cancer Institute, Buffalo, New York, United States of America; Health Canada, Canada

## Abstract

**Background:**

Endometrial cancer is the most common gynecologic malignancy in developed countries and little is known about the underlying mechanism of stage and disease outcomes. The goal of this study was to identify differentially expressed genes (DEG) between late vs. early stage endometrioid adenocarcinoma (EAC) and uterine serous carcinoma (USC), as well as between disease outcomes in each of the two histological subtypes.

**Methodology/Principal Finding:**

Gene expression profiles of 20 cancer samples were analyzed (EAC = 10, USC = 10) using the human genome wide illumina bead microarrays. There was little overlap in the DEG sets between late vs. early stages in EAC and USC, and there was an insignificant overlap in DEG sets between good and poor prognosis in EAC and USC. Remarkably, there was no overlap between the stage-derived DEGs and the prognosis-derived DEGs for each of the two histological subtypes. Further functional annotation of differentially expressed genes showed that the composition of enriched function terms were different among different DEG sets. Gene expression differences for selected genes of various stages and outcomes were confirmed by qRT-PCR with a high validation rate.

**Conclusion:**

This data, although preliminary, suggests that there might be involvement of distinct groups of genes in tumor progression (late vs. early stage) in each of the EAC and USC. It also suggests that these genes are different from those involved in tumor outcome (good vs. poor prognosis). These involved genes, once clinically verified, may be important for predicting tumor progression and tumor outcome.

## Introduction

Endometrial cancer is the most common gynecologic malignancy in developed countries, including an estimated 42,160 new cases in the United States in 2009 and claiming almost 7,780 lives [1]. Based on clinico-pathologic and molecular data, endometrial adenocarcinomas are dichotomized into two types: type I, endometrioid adenocarcinoma (EAC) and mucinous adenocarcinoma; type II, uterine serous carcinoma (USC) and clear cell carcinoma (CCC) [2]. EACs are the most frequent subtype and account for more than 80% of all endometrial adenocarcinomas. They are associated with obesity, exogenous hormonal therapy and they tend to present as low grade, early stage tumors with good outcomes, often cured with surgery alone. However, approximately 11% to 16% of women with EAC will present with FIGO (International Federation of Gynecology and Obstetrics) stage II, III and stage IV disease with 5-year survival rate of 70%, 40–50% and 15–20% respectively. USCs account for 3–10% of endometrial carcinomas. While USCs represent a minority of total endometrial cancer cases they are responsible for a disproportionate number of deaths [3], [4]. They are high grade tumors with deep myometrial invasion and lymphovascular involvement [5]. The 5-year survival rates are estimated to be 50% for stage II, 20% for stage III and 5–10% for stage IV disease [6].

Molecular genetic data supports the idea that endometrial carcinomas are likely to develop through a multi-step process of oncogene activation and tumor suppressor gene inactivation. In addition, studies have demonstrated that molecular alterations are specific for type I and type II endometrial carcinoma. Type I cancer is characterized by mutation of *PTEN*, and defects in DNA mismatch repair (as evidenced by the microsatellite instability phenotype) [7], [8]. Type II cancers are characterized by p53 and Her-2/neu mutations [9], [10]. However, these gene alterations alone do not explain the different behavior and outcomes in type I and type II cancers. Most of the studies using cDNA microarray analysis have only focused on defining differential gene expression among different histologic types of endometrial cancer [11]–[17]. The aims of this study were focused on stages and outcomes in two histologic types: EAC and USC. The purpose was to identify the difference in gene expression patterns in late stages (stage III and IV) versus early stages (stage I and II) in each of the two types and in patients with good prognosis versus poor prognosis in EAC and USC, respectively.

## Results

### Comparison of Stages and Outcomes

Under the framework of linear model, we inferred the differential expression based on the following collected patients' characteristics: EAC stage (late n = 5 *vs.* early n = 5), USC stage (late n = 5 *vs.* early n = 5), EAC prognosis (good n = 6 *vs.* poor n = 4), and USC prognosis (good n = 6 *vs.* poor n = 4). The number of identified DEGs and the subgroup restricted by desired fold change for each comparison are illustrated in Table S1. A direct comparison of their total gene expression patterns was performed to evaluate the differences among each category.

**Figure 1:**
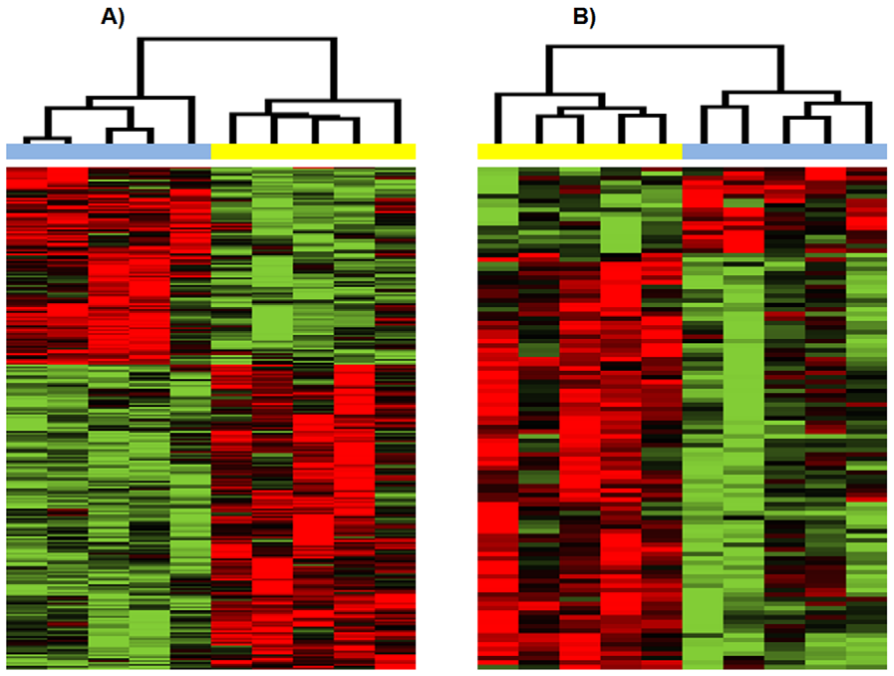
Hierarchical clustering of patient samples based on differentially expressed genes (P<0.01) obtained from comparing late stage versus early stage in the USC group and EAC group, respectively. *A) USC group*. *B) EAC group*. In clustering dendrogram, blue stands for early stage samples while yellow stands for late stage samples. Red means up-regulated while green means down regulated.

Hierarchical clustering of patients samples based on DEGs (p<0.01) obtained from comparing late vs. early stage in USC group and EAC group, respectively, is illustrated in Figure 1. We identified 274 DEGs at significance level (p<0.01) in patients with USC, with 165 genes up-regulated and 109 genes down-regulated in late stage disease. The 274 DEGs separate the 5 USC late stage patients from the 5 USC early stage patients. For stage comparison (late vs. early) in patients with EAC, we identified 111 significant DEGs (p<0.01), with 92 genes up-regulated and 19 genes were down-regulated in late stages. The 111 DEGs accurately separate the 5 EAC late stage patients from the 5 EAC early stage patients.

**Figure 2:**
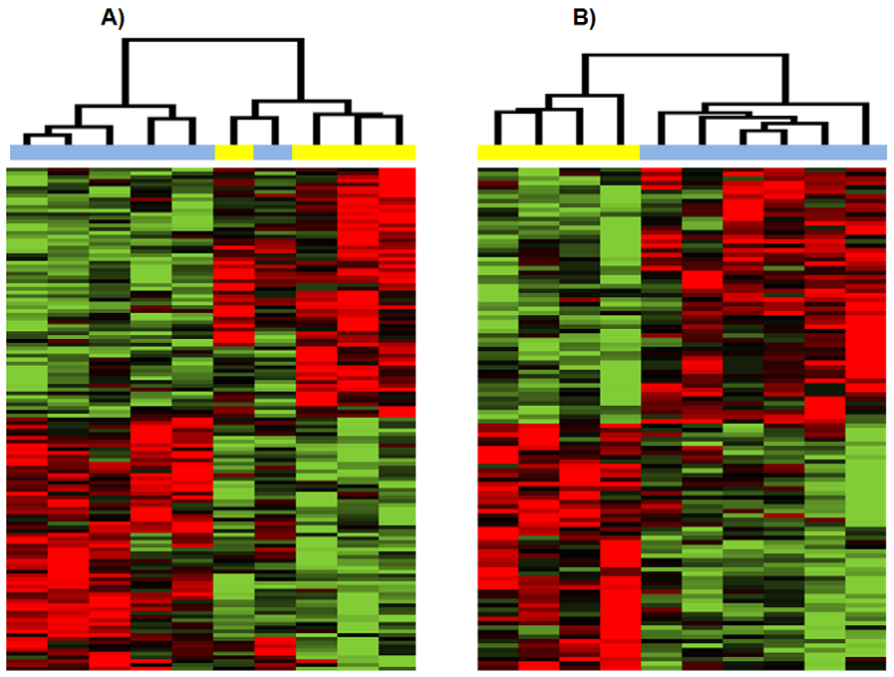
Hierarchical clustering of patient samples based on differentially expressed genes (P<0.01) obtained from comparing good prognosis versus poor prognosis in the USC group and EAC group, respectively. *A) USC group*. *B) EAC group*. In clustering dendrogram, blue stands for good prognosis samples while yellow stands for poor prognosis samples. Red means up-regulated while green means down regulated.

For prognosis comparison (good vs. poor), we identified 135 and 112 DEGs at a significance level (p<0.01), for USC and EAC respectively (Figure 2). The 112 DEGs derived from good vs. poor prognosis comparison in EAC subtype accurately separate the 6 EAC good prognosis patients from the 4 EAC poor prognosis patients. Although less perfect, the 135 DEGs derived from good vs. poor prognosis comparison in USC subtype can separate 5 of the 6 USC good prognosis patients from the 4 USC poor prognosis patients.

The complete list of DEGs with at least 2-fold change from the four separate comparisons described above is listed in Table S2–S5.

**Figure 3:**
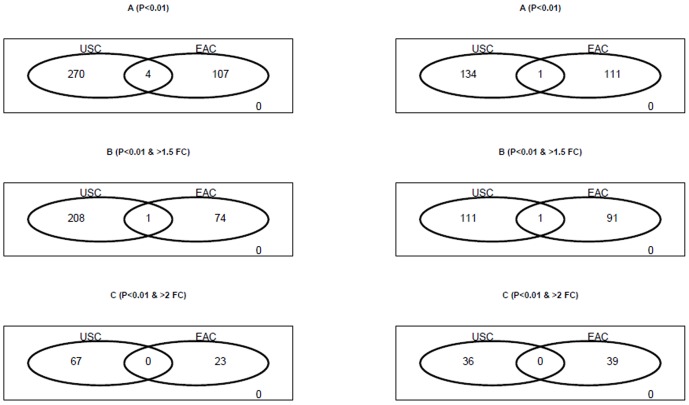
Venn diagrams show little overlap for DEGs derived from the USC and EAC groups, respectively. *Left)* DEGs from comparing late stage versus early stage in the USC group and EAC group, respectively. *Right)* DEGs from comparing good prognosis versus poor prognosis in the USC group and EAC group, respectively. A) DEGs as defined by P-value<0.01. B) DEGs with at least 1.5-fold change. C) DEGs with at least 2-fold change.

To compare the tumor progression mechanism level between different endometrial adenocarcinoma subtypes (USC vs. EAC) at the transcriptome level, we first examined the overlap between stage-derived DEGs in USC and stage-derived DEGs in EAC. As shown in Figure 3, we found that there is minimal overlap between the two DEG sets. Only 4 DEGs are shared by the 274 stage-derived DEGs in USC and the 111 stage-derived DEGs in EAC. There is no overlap when two-fold change is included as the restriction.

We then evaluated the overlap between prognosis-derived DEGs in USC and prognosis-derived DEGs in EAC. As shown in Figure 3 we found that there is minimal overlap between the two DEG sets. Only 1 DEG is shared by the 135 prognosis-derived DEGs in USC and the 112 prognosis-derived DEGs in EAC.

**Figure 4:**
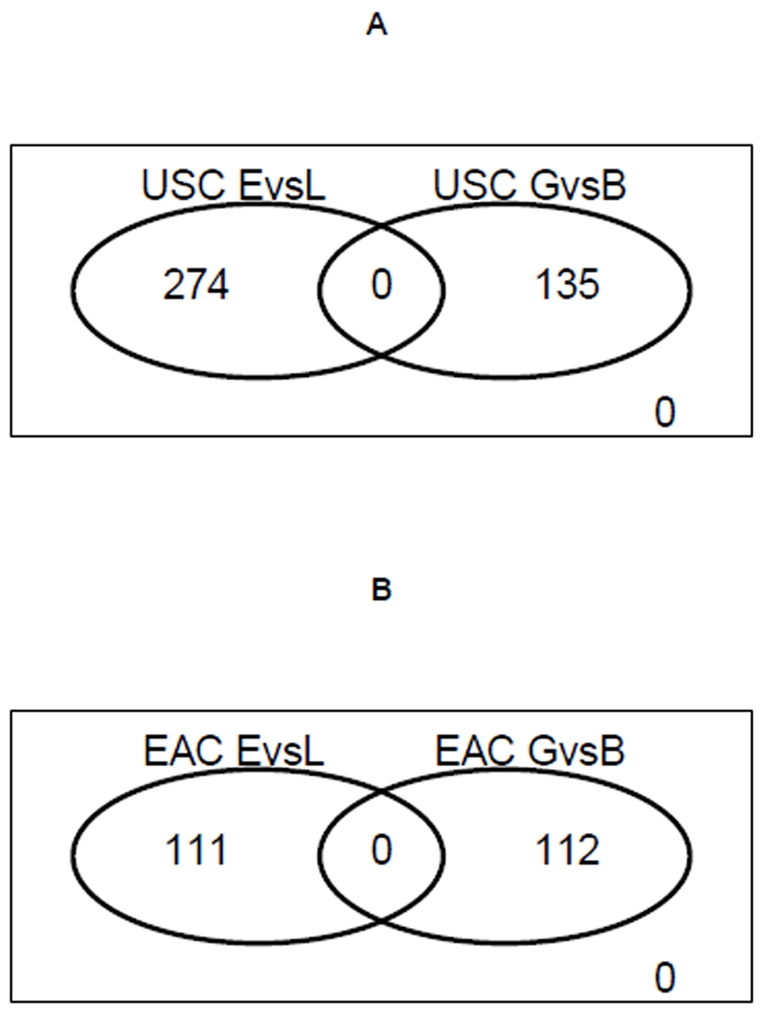
Venn diagrams show no overlap between stage-related DEGs (late versus early) and outcome-related DEGs (good versus poor) in both USC and EAC groups. *EvL* means DEGs from comparing late stage versus early stage patients. *GvB* means DEGs from comparing good prognosis versus poor prognosis patients. A) DEGs as defined by P-value<0.01 in USC group. B) DEGs as defined by P-value<0.01 in EAC group.

Figure 4 illustrates the overlap between stage-derived DEGs and prognosis-derived DEGs in both USC and EAC subtypes. Remarkably, we found no overlap between stage-derived DEGs and prognosis-derived DEGs in both subtypes. For USC, the 274 stage-derived DEGs are distinct from the 135 prognosis-derived DEGs, and for EAC, the 111 stage-derived DEGs are distinct from the 112 prognosis-derived DEGs.

The lack of overlap between stage-related (late vs. early) and prognosis-related (good vs. poor) DEG sets in each subtype is of particular interest. This suggests that it might be necessary to identify and develop separate diagnosis biomarkers for endometrial adenocarcinoma stage diagnosis and patient outcome prediction.

**Figure 5:**
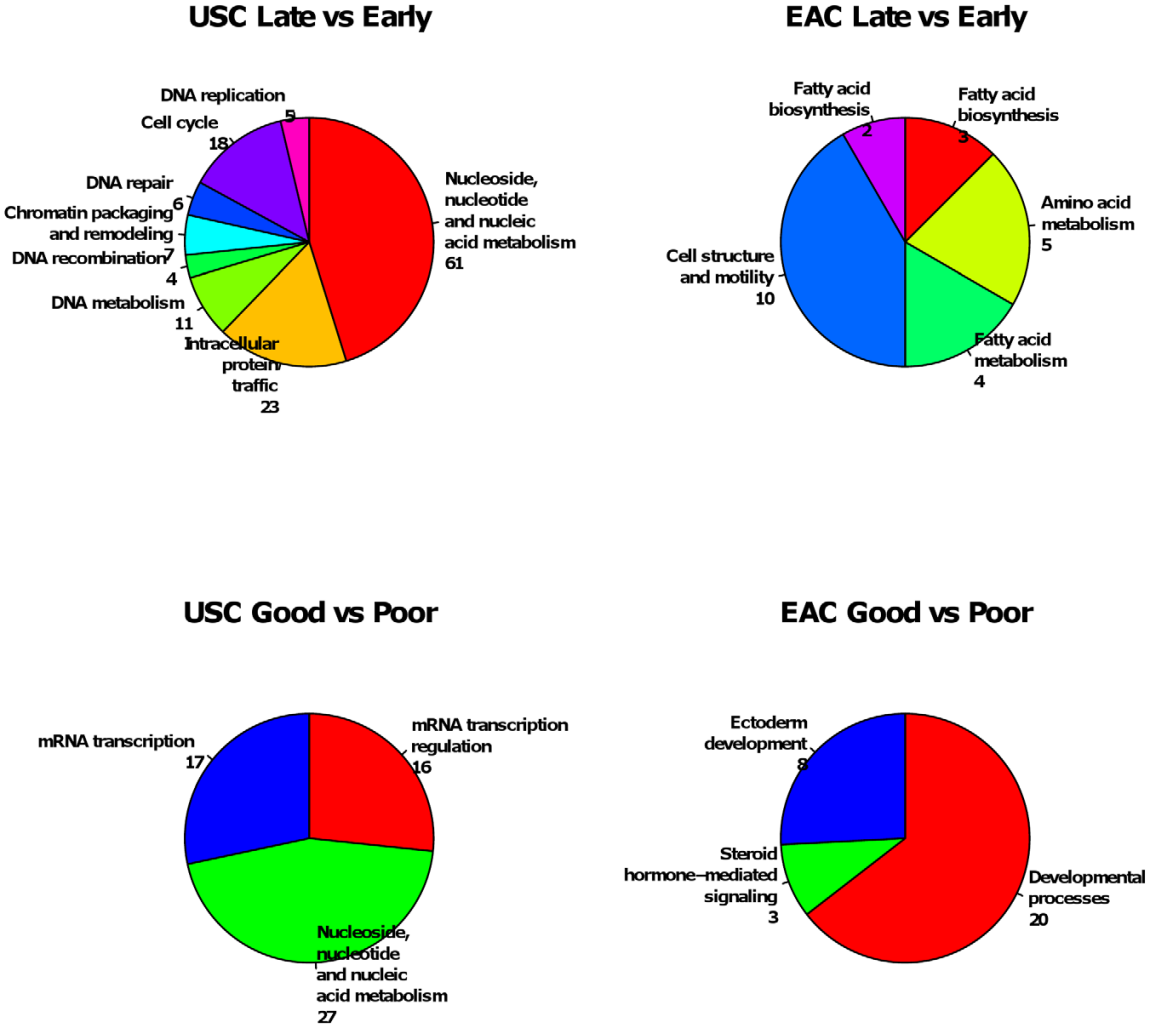
Enriched function annotation of differentially expressed genes (P<0.01) identified by microarray. Enriched functional annotations are reported by NCBI DAVID API server with default setting. The number following each enriched functional term is the number of annotated DEGs. *A*) Enriched functional annotation for stage-related DEGs (late versus early) in USC group. *B*) Enriched functional annotation for stage-related DEGs in EAC group. *C*) Enriched functional annotation for outcome-related DEGs (good versus poor) in USC group. *D*) Enriched functional annotation for outcome-related DEGs in EAC group.

Further functional annotation of DEGs showed that the composition of enriched function terms were different between the four DEG sets (Figure 5). For stage-related DEGs, the most enriched function terms in USC group are nucleic metabolism, protein traffic and cell cycle, while the most enriched function terms in EAC group are cell structure and mobility, amino acid metabolism and fatty acid metabolism. For outcome-related DEGs, the most enriched function terms in USC group are nucleic metabolism and mRNA transcription, while the most enriched function terms in EAC group are developmental process. Remarkably, the only overlapped function term is nucleic metabolism which is enriched in both stage-related and outcome-related DEGs of USC group. This suggests that different tumor histology, stage and disease outcomes in endometrial adenocarcinoma might involve either different pathways or different components of common pathways. However, these results should be interpreted with caution given the relatively small sample size, and a larger group in future study will be needed for validations.

### qRT-PCR Validation of Microarray Data

**Figure 6:**
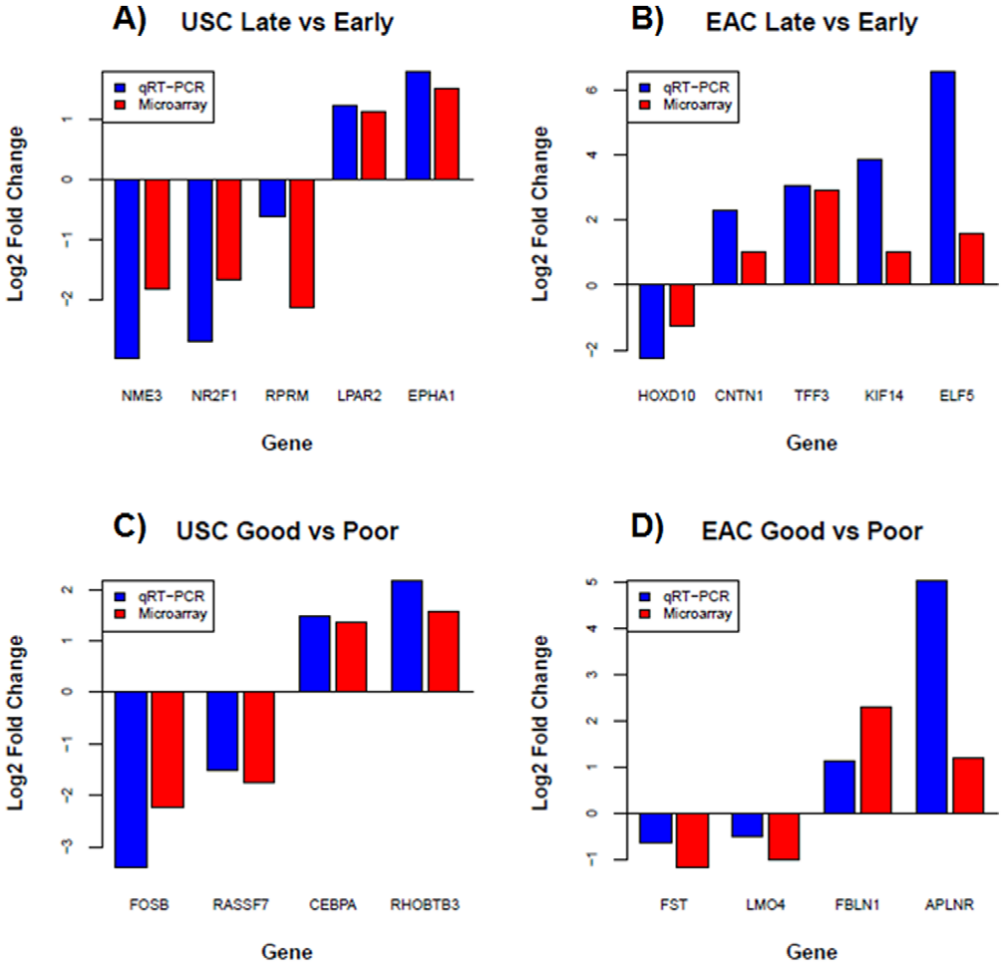
qRT-PCR validation of differentially expressed genes (P<0.01) with at least two-fold expression change as identified by microarray. *A*) stage-related DEGs (late versus early) in USC group. *B*) stage-related DEGs in EAC group. *C*) Outcome-related DEGs (good versus poor) in USC group. *D*) Outcome-related DEGs in EAC group. Blue bar is the fold change estimated by qRT-PCR, while red bar is the fold change estimated by microarray. The fold change is shown in log2 scale (i.e., >1 means at least 2-fold up, while <−1 means at least 2-fold down). The three genes with less than two-fold change in expression level based on qRT-PCR are *FST, LMO4* and *RPRM*.

We randomly selected 18 differentially expressed genes with at least 2-fold change identified by microarray for validation by quantitative Real Time PCR (qRT-PCR). Selected genes include those up-regulated in late vs. early USC (*LPAR2 and EPHA1*), as well as in EAC *(CNTN1, ELF5, KIF14 and TFF3)* and those they were down-regulated in late vs. early USC (*RPRM, NME3, NR2F1*), as well as in EAC (*HOXD10*). The selected genes also include those up-regulated in good vs. poor diagnosis in USC *(RHOBTB3, CEBPA)* as well as in EAC (*FBLN1, APLNR*), and those down-regulated in good vs. poor diagnosis in USC (*FOSB, RASSF7*) as well as in EAC (*FST, LMO4*). qRT-PCR data of 15 genes indicated at least 2-fold change in expression levels and were concordant with the microarray data, yielding a validation rate of 15/18. Three genes, *FST, LMO4* and *RPRM*, have less than 2-fold change in expression level based on qRT-PCR. However, their directions of expression change estimated by qRT-PCR are consistent with those estimated from microarray (Figure 6).

## Discussion

The main focus of the study is to identify and evaluate gene expression patterns in late vs. early stage and good vs. poor prognosis in each of endometrioid and serous types. Using the Illumina HumanHT-12 v3 microarray, we found 274 and 111 stage-related DEG in each of USC and EAC respectively. However, we were not able to find any overlap for DEGs with at least 2 fold changes in late stages EAC and USC versus early stages EAC and USC, indicating that tumor progression of different endometrial adenocarcinoma subtypes might be characterized by distinct gene expression signatures. In addition, we found 112 DEGs that are differently expressed for good vs. poor prognosis in EAC and 135 DEGs in USC. Only 1 DEGs is shared by the 135 prognosis-derived DEGs in USC and the 112 prognosis-derived DEGs in EAC. These findings indicate that tumor outcome of different endometrial adenocarcinoma subtypes might also characterized by distinct gene expression signatures. In other words, DEGs derived from USC might exclusively contain USC-specific prognosis biomarkers, while the DEGs derived from EAC might exclusively contain EAC-specific prognosis biomarkers. This confirms the existence of a distinct gene expression signature between endometrioid and serous carcinoma as previously seen [11], [15], [16], and that there is a distinct gene expression signature driving late vs. early stages in each of these two types. Additionally, the lack of overlap between stage-related and prognosis-related DEG sets in each subtype is of particular interest suggesting genes that drive and control stages might be distinct from the genes that drive and control outcomes. As a result, it would be of paramount interest to identify tailored biomarkers for outcome prediction and treatment modalities in patients with endometrial adenocarcinoma. Clearly, given the relatively small sample size, these findings should be interpreted with caution and a larger cohort is needed to validate these findings.

Reviewing the microarray data on endometrial adenocarcinomas, there were a few DEGs that have previously been described in literatures and confirmed in this study [11]–[17]. For example, *Ephrin receptor A1 (EphA1*), located at 7q32-q36, is a novel receptor tyrosine kinase gene. The EphA1receptor/ephrin ligand system has been implicated in tumor progression in a number of malignancies where they are strongly involved in tumorigenesis including metastatsis, angiogenesis and invasion [11], [18]–[20]. *Trefoil factor 3 (TFF3)*, located at 21q22.3, belongs to a family of small mucin-associated polypeptides that can regulate cancer progression by increasing tumor metastasis [16], [17], [21], [22]. *E74-like factor 5 (ELF5)* or *epithelium –specific ETS factor 2 (ESE2)*, located at 11p15-p13, is a member of the ETS family of transcription factors and has been implicated to play a key role in cell proliferation, differentiation, apoptosis and tumorigenesis [17], [23].

However, a number of the DEGs identified in our study represent novel ones, not captured by previous studies. For example, l*ysophosphatidic acid receptor 2 (LPAR2)*, mapped at 19p12 locus, is an important extracellular signaling molecule that mediates a wide range of actions such as cell proliferation, cell survival, migration, adhesion, and angiogenesis. Recently, *LAPR2* was found to be overexpressed in ovarian tumors and authors have speculated that this gene may contribute to the initiation, progression and even aggressive tumor behavior [24], [25]. Based on our data, LAPR2 could be possibly be an indicator of late stage USC and thus causing aggressive tumor behavior. Other novel DEGs that were found to be over-expressed in late vs. early EAC are *Contactin 1 (CNTN1*) at 12q11-q12 and *kinesin family member 14 (KIF14) or KIAA0042* mapped at 1q31 locus. *Contactin- 1* is a metastasis promoter gene that plays an essential role in tumor metastasis and tumor invasion. In animal studies, knockdown contactin-1 resulted in inhibition of tumor metastasis and an increase in survival. In patients with lung adenocarcinoma, high *Contactin-1* expression was directly correlated with tumor stage, lymph node metastasis and poor survival [29]. Minimal literature has been published regarding its mechanism; therefore, inhibitors of *contactin-1* could be a possible target therapy in advanced stage EAC. Over-expression of *KIF14* by RT-PCR was seen in retinoblastoma and numerous other cancer types including breast, lung, larynx, and hepatocellular carcinoma where numerous studies suggested that *KIF14* might have oncogenic potential [30]–[32]. Although its cellular function is not clear, *KIF14* belongs to the kinesin family and it usually plays an important role in mitosis. Its expression was a predictor of tumor grade and a decreased disease-free survival rate in breast cancer [33]. In our study, the over-expression of *KIF14* is an indicator of advanced, late stage EAC. Among the genes under-expressed in late vs. early stages USC, we found nonmetastatic cells 3 (*NME3*) located at 16q13, and nuclear receptor subfamily 2 or transcription factor COUP-1 *(NR2 F1/ COUP- TF1)* gene, which is mapped at 5q14 locus. *NME3* is a member of the *nm23* putative suppressor gene family associated with metastasis, differentiation and apoptosis of cancer cells [26]. In our study, the finding of *NME3* under-expression in late stage USC might be interpreted as evidence of it functions as a metastasis suppressor. *NR2F1*, also known as *COUP TF1*, is chicken ovalbumin upstream promoter transcription factor (member of the orphan steroid receptor superfamily). Studies showed the involvement of *COUP-TF1* in cell differentiation and growth in endometrial and ovarian cancer cells [27]. Recently, this gene was found to play a role in lymphangeogenesis via regulation of the vascular endothelial growth factor (VEGF) in cancer [28]. *HOXD10* belongs to the HOX regulatory family of genes that encode transcription factors which are essential during embryonic development [34]. *HOX* genes are important for human endometrial development and receptivity. *HOXD10* was found to be strongly expressed in normal human uterine tissue. The expression of *HOXD10* was extremely reduced in endometrial carcinoma especially in high grade tumors, suggesting that it could have a role in oncogenesis [35].

RHO-related BTB domain-containing protein3 *(RHOBTB3)* or also known as *KIAA0878* has been mapped on 5q14.3 locus and the gene has been differentially expressed and confirmed by qRT-PCR in good vs.poor USC outcome. *RhoBTB3* is a member of the *RHOBTB* subfamily of Rho GTPases that play a role in mediating cell size, proliferation, apoptosis, survival, polarity, call adhesion and membrane trafficking [36]. Recent studies have suggested that *RhoB* is involved in tumor suppression. These studies suggested that *RhoB* was detected in normal tissue yet its expression was dramatically lost during cancer progression in lung and head and neck squamous cell carcinoma [37], [38]. In line with these findings, high expression of *RhoB* was associated with favorable outcome in bladder cancer. In our study, we suggested that *RhoBTB3* might serve as a potential tumor marker for good prognosis in USC. RAS association domain family 7 (*RASSF7*) is located at 11p15.5 and it belongs to the Ras-domain family of ten members that are implicated in various cellular mechanisms including apoptosis, cell cycle control, and microtubule stabilization [39]. They are down-regulated by epigenetic mechanisms, indicating the potential role of a tumor suppressor gene. However, this does not currently exist in *RASSF7* [40]. Recently *RASSF7* was found in numerous tissues and knocking down *RASSF*7 function resulted in blocking spindle formation, triggering a mitotic arrest, nuclear breakdown and apoptosis. This suggests the possibility that *RASSF7* could have a role in promoting cancer cell development [41]. In our study the under-expression of *RASSF7* in USC correlated with good prognosis and the detection of RASSF7 silencing by methylation study could have potential clinical use for USC prognosis and treatment. Finally, Fibulin1 *(FBLN1)*, mapped on 22q13.3 gene, belongs to a family of secreted glycoproteins. Fibulin family has been shown to modulate cell morphology, growth, adhesion and motility. In particular, *FBLN1* appeared to have a role in inhibiting cell adhesion, spreading, motility and invasion in human cancer cells [42]. *In vivo* studies showed increased *FBLN1* expression in ovarian and breast carcinomas. Others had showed its down-regulation in prostate and gastric cancer [43]. Therefore, speculation still exists regarding *FBLN1* as a tumor-suppressor gene or an oncogene or it might even have dual functions [44]. In our study, the over-expression of the *FBLN1* protein was observed for good prognosis in EAC.

One limitation of this study is the relatively small sample size which does not provide us enough power for statistical analysis of expression levels of DEGs and clinical characteristics. The result should be interpreted with caution because of the small sample size and undetermined molecular mechanisms of novel DEGs. Nevertheless, novel DEGs found in our studies, once narrowed down and verified in future studies with larger cohort, might have potential prognostic and therapeutic effects in each of EAC and USC.

In conclusion, although the sample size was small for a definite conclusion, we believe that our findings shed meaningful insights into the clinical study of endometrial cancer patients that warrant further investigation. Future large studies and advanced technologies are warranted to confirm our findings and further explore the potential of DEGs to be utilized clinically as novel biomarkers for endometrial cancer.

## Materials and Methods

### Tissue Specimens

Flesh-frozen cancer specimens were obtained from 20 patients undergoing surgery for uterine cancer at Roswell Park Cancer Institute (RPCI) including 10 cases of EAC, and 10 cases of USC. Five out of 10 EAC specimens were from patients with early stage disease (FIGO stage I and II), and five specimens were from patients with late stage (FIGO stage III and IV). Of these 5 early stage EAC, 3 had good outcome and 2 had poor outcome. Similarly, 3 late stage EAC had a good prognosis and 2 had a poor prognosis. The same patient distribution was for USC cases. All of the tissue samples were collected under an Institutional Review Board-approved protocol at RPCI. The hematoxylin-eosin (HE) slides were reviewed by one Pathologist to confirm the tumor subtype and FIGO grade. All patients were treated per National Comprehensive Cancer Network guidelines [45]. Patients' charts were reviewed for patient follow-up which ranged from 18 months to 60 months. Good prognosis is defined as patients who are alive with no evidence of disease at last follow-up. Poor prognosis is defined as patients who are alive with recurrent disease, persistence, or progression of disease as well as patients who died from disease. The Health Sciences Institutional Review Board (HSIRB) of Roswell Park Cancer Institute has authorized this research.

### Sample Processing and Gene Expression Profiling

#### RNA preparation

The fresh frozen tissues were cut and examined to make certain that the tissue contains >80% tumor. Total RNA from 10–20 mg fresh frozen tissues were prepared using the RNeasy midi kits (Qiagen, Valencia, CA) following manufacturer's instructions. After elution, RNA samples were concentrated by EtOH precipitation at −20°C overnight, and resuspended in nuclease-free water. Before labeling, RNA samples were quantitated using a ND-1000 spectrophotometer (NanoDrop Wilmington, DE) and evaluated for degradation using a 2100 Bioanalyzer (Agilent Technologies, Santa Clara, CA). Samples were required to have a RIN >6.5, an OD 260∶280 of 1.9–2.1, an OD 260/230 >1.5 and >1.5 28S:18S ratio of the ribosomal bands for gene expression array analysis.

#### Gene expression assay

Expression profiling was accomplished using the HumanHT-12 v3 whole-genome gene expression direct hybridization assay (Illumina, San Diego, CA) as previously published [46]. Each array contains full-length 50-mer probes representing more than 48,000 well-annotated RefSeq transcripts, including >25,400 unique, curated, and up-to-date genes derived from the National Center for Biotechnology Information Reference Sequence (NCBI RefSeq) database (Build 36.2, Release 22). Initially, 250 ng total RNA was converted to cDNA, followed by an in vitro transcription step to generate labeled cRNA using the Ambion Illumina Total Prep RNA Amplification Kit (Ambion, Austin, TX) as per manufacturer's instructions. The labeled probes were then mixed with hybridization reagents and hybridized overnight to the HumanHT-12 v3 BeadChips. Following washing and staining, the BeadChips were imaged using the Illumina BeadArray Reader to measure fluorescence intensity at each probe. The intensity of the signal corresponds to the quantity of the respective mRNA in the original sample. The expression profiles have been deposited in NCBI's Gene Expression Omnibus (GEO) with GSE accession number GSE23518.

#### Data analysis

BeadChip data files are analyzed with Illumina's GenomeStudio gene expression module and R-based Bioconductor package to determine gene expression signal levels [47]. Briefly, the raw intensity of Illumina Human HT-12 v3.0 gene expression array was scanned and extracted using BeadScan, with the data corrected by background subtraction in GenomeStudio module. The *lumi* module in the R-based Bioconductor Package was used to transform the expression intensity into *log2* scale [48]. The log2 transformed intensity data were normalized using Quantile normalization function.

We used the *Limma* program in the R-based *Bioconductor* package to calculate the level of differential expression [49]. Briefly, a linear model was fit to the data (with cell means corresponding to the different conditions and a random effect for array), and the list of differentially expressed genes (DEGs) with *Pvalue*<0.01 were obtained by performing the following comparisons based on collected patients' characteristics: USC stage (late *vs.* early), EAC stage (late *vs.* early), USC prognosis (good *vs.* poor), and EAC prognosis (good *vs.* poor).

Following single gene-based significance testing, we used the expression value of DEGs (*Pvalue*<0.01) to cluster the patients for each comparison. Our purpose was to determine whether the identified DEGs for each comparison are able to serve as potential gene signature to classify patients into their corresponding clinicopathologic groups. Hierarchical clustering algorithm based on the average linkage of Pearson Correlation was employed [50]. The DEGs were analyzed for enriched biological process terms using the NCBI DAVID server (http://david.abcc.ncifcrf.gov) with default setting [51]. All calculations were carried out under R statistics computing.

#### Quantitative real time PCR analysis

The expression level of 18 genes *APLNR*, *CEBPA*, *CNTN1*, *ELF5, EPHA1, FBLN1, FOSB, FST, HOXD10, KIF14, LMO4, LPAR2, NME3, NR2F1, RASSF7, RHOBTB3, RPRM, TFF3* selected for validation was determined using Taqman qRT-PCR gene expression Assay On Demand Probe/Primers (Applied Biosystems, Foster City, CA), with housekeeping gene GAPDH as an endogenous control. Samples were run on the AB HT7900 Sequence Detection System according to default parameters, with three replicate assays for each gene in each sample. Using the RQ Manager Software 2.2.2 (AB, Foster City, CA) the data was analyzed and the baseline and the threshold were verified for each gene of interest. qRT-PCR data were the normalized expression values in which the housekeeping gene GAPDH was used as the reference gene. For each assay, the average GAPDH Ct (Cycle threshold) value in the TaqMan qPCR assay was subtracted from the Ct of gene of interest to obtain a ΔCt value (gene of interest - GAPDH).

## Supporting Information

Table S1 Summary of the number of DEGs obtained from four separate comparisons based on patients' Clinicopathologic data. (DOC)

Table S2 The list of DEGs with at least two-fold change obtained from comparisons of late stage vs. early stage in USC group. (DOC)

Table S3 The list of DEGs with at least two-fold change obtained from comparisons of late stage vs. early stage in EAC group. (DOC)

Table S4 The list of DEGs with at least two-fold change obtained from comparisons of good prognosis vs. poor prognosis in USC group. (DOC)

Table S5 The list of DEGs with at least two-fold change obtained from comparisons of good prognosis vs. poor prognosis in EAC group. (DOC)
