# Supplementary material for: Microarray Analysis Reveals Distinct Gene Expression Profiles Among Different Tumor Histology, Stage and Disease Outcomes in Endometrial Adenocarcinoma
Source: PLoS One. 2010 Nov 8;5(11):e15415. doi: 10.1371/journal.pone.0015415 (PMC2975707; doi:10.1371/journal.pone.0015415)
Supplement: Table S1 — Summary of the number of DEGs obtained from four separate comparisons based on patients' Clinicopathologic data. (DOC) [file pone.0015415.s001.doc]

**Tabel S1** Summary of the number of DEGs obtained from four separate comparisons based on patients’ Clinicopathologic data.

|  | **USC**  **(Late vs. Early)** | **EAC**  **(Late vs. Early)** | **USC**  **(Good vs. Poor)** | **EAC**  **(Good vs. Poor)** |
| --- | --- | --- | --- | --- |
| **# of Patients** | 5 vs. 5 | 5 vs. 5 | 6 vs. 4 | 6 vs. 4 |
| **# of DEGs (P<0.01)** | 274  (165^/109*) | 111  (92/19) | 135  (68/67) | 112  (57/55) |
| **# of DEGs with**  **>= 1.5-fold change** | 209  (115/94) | 75  (60/15) | 112  (57/55) | 92  (50/42) |
| **# of DEGs with**  **>= 2-fold change** | 67  (19/48) | 23  (19/4) | 36  (19/17) | 39  (23/16) |

^: Up-regulated.

*: Down-regulated.
