# Supplementary material for: Microarray Analysis Reveals Distinct Gene Expression Profiles Among Different Tumor Histology, Stage and Disease Outcomes in Endometrial Adenocarcinoma
Source: PLoS One. 2010 Nov 8;5(11):e15415. doi: 10.1371/journal.pone.0015415 (PMC2975707; doi:10.1371/journal.pone.0015415)
Supplement: Table S2 — The list of DEGs with at least two-fold change obtained from comparisons of late stage vs. early stage in USC group. (DOC) [file pone.0015415.s002.doc]

**Table S2.**The list of DEGs with at least two-fold change obtained from comparisons of late stage vs. early stage in USC group.

1. Up-regulated genes

| **Illumina ID** | **Log2 FC** | **P.Value** | **ENTREZ** | **SYMBOL** | **Description** |
| --- | --- | --- | --- | --- | --- |
| **WDR72** | 2.19 | 0.003239 | 256764 | WDR72 | WD repeat domain 72 (WDR72), mRNA. |
| **FLJ43692** | 1.86 | 0.000974 | 445328 | FLJ43692 | ARHGEF5-like (FLJ43692), mRNA. |
| **UBB** | 1.83 | 0.000447 | 7314 | UBB | ubiquitin B (UBB), mRNA. |
| **LRP4** | 1.53 | 0.00817 | 4038 | LRP4 | low density lipoprotein receptor-related protein 4 (LRP4), mRNA. |
| **EPHA1*** | 1.51 | 0.001363 | 2041 | EPHA1 | EPH receptor A1 (EPHA1), mRNA. |
| **GPT2** | 1.46 | 0.005674 | 84706 | GPT2 | glutamic pyruvate transaminase (alanine aminotransferase) 2 (GPT2), mRNA. |
| **ZNF91** | 1.37 | 0.000135 | 7644 | ZNF91 | zinc finger protein 91 (ZNF91), mRNA. |
| **ATF5** | 1.36 | 0.001375 | 22809 | ATF5 | activating transcription factor 5 (ATF5), mRNA. |
| **RAB3IP** | 1.31 | 0.002094 | 117177 | RAB3IP | RAB3A interacting protein (rabin3) (RAB3IP), transcript variant beta 1, mRNA. |
| **SOX4** | 1.27 | 0.002103 | 6659 | SOX4 | SRY (sex determining region Y)-box 4 (SOX4), mRNA. |
| **EDG4*** | 1.14 | 0.004126 | 9170 | EDG4 | endothelial differentiation, lysophosphatidic acid G-protein-coupled receptor, 4 (EDG4), mRNA. |
| **FBN3** | 1.12 | 2.56E-05 | 84467 | FBN3 | fibrillin 3 (FBN3), mRNA. |
| **PRSS1** | 1.08 | 0.002227 | 5644 | PRSS1 | protease, serine, 1 (trypsin 1) (PRSS1), mRNA. |
| **ASNS** | 1.06 | 0.004212 | 440 | ASNS | asparagine synthetase (ASNS), transcript variant 1, mRNA. |
| **PLAGL2** | 1.05 | 0.006447 | 5326 | PLAGL2 | pleiomorphic adenoma gene-like 2 (PLAGL2), mRNA. |
| **NCAPD2** | 1.04 | 0.008453 | 9918 | NCAPD2 | non-SMC condensin I complex, subunit D2 (NCAPD2), mRNA. |
| **HDAC2** | 1.01 | 0.001417 | 3066 | HDAC2 | histone deacetylase 2 (HDAC2), mRNA. |
| **TMEM14B** | 1.00 | 0.001666 | 81853 | TMEM14B | transmembrane protein 14B (TMEM14B), mRNA. |
| **FOXP4** | 1.00 | 0.000211 | 116113 | FOXP4 | forkhead box P4 (FOXP4), transcript variant 1, mRNA. |

1. Down-regulated genes

| **Illumina ID** | **Log2 FC** | **P.Value** | **ENTREZ** | **SYMBOL** | **Description** |
| --- | --- | --- | --- | --- | --- |
| **REG1A** | -3.65 | 0.000288 | 5967 | REG1A | regenerating islet-derived 1 alpha (pancreatic stone protein, pancreatic thread protein) (REG1A), mRNA. |
| **C2ORF40** | -2.86 | 0.000426 | 84417 | C2orf40 | chromosome 2 open reading frame 40 (C2orf40), mRNA. |
| **CRYAB** | -2.26 | 0.002937 | 1410 | CRYAB | crystallin, alpha B (CRYAB), mRNA. |
| **OLFML1** | -2.18 | 4.47E-05 | 283298 | OLFML1 | olfactomedin-like 1 (OLFML1), mRNA. |
| **SRPX** | -2.15 | 0.00016 | 8406 | SRPX | sushi-repeat-containing protein, X-linked (SRPX), mRNA. |
| **RPRM*** | -2.14 | 0.002236 | 56475 | RPRM | reprimo, TP53 dependent G2 arrest mediator candidate (RPRM), mRNA. |
| **GNG11** | -2.12 | 0.00044 | 2791 | GNG11 | guanine nucleotide binding protein (G protein), gamma 11 (GNG11), mRNA. |
| **ZCCHC24** | -2.08 | 0.001345 | 219654 | ZCCHC24 | zinc finger, CCHC domain containing 24 (ZCCHC24), mRNA. |
| **COL6A1** | -1.98 | 0.004257 | 1291 | COL6A1 | collagen, type VI, alpha 1 (COL6A1), mRNA. |
| **PLAC9** | -1.86 | 0.007686 | 219348 | PLAC9 | placenta-specific 9 (PLAC9), mRNA. |
| **PPP1R3C** | -1.86 | 0.004459 | 5507 | PPP1R3C | protein phosphatase 1, regulatory (inhibitor) subunit 3C (PPP1R3C), mRNA. |
| **NME3*** | -1.83 | 0.000109 | 4832 | NME3 | non-metastatic cells 3, protein expressed in (NME3), mRNA. |
| **JAM2** | -1.83 | 0.008703 | 58494 | JAM2 | junctional adhesion molecule 2 (JAM2), mRNA. |
| **SNAI2** | -1.82 | 0.00037 | 6591 | SNAI2 | snail homolog 2 (Drosophila) (SNAI2), mRNA. |
| **CAMK2N1** | -1.79 | 0.007629 | 55450 | CAMK2N1 | calcium/calmodulin-dependent protein kinase II inhibitor 1 (CAMK2N1), mRNA. |
| **HSPB7** | -1.77 | 0.008678 | 27129 | HSPB7 | heat shock 27kDa protein family, member 7 (cardiovascular) (HSPB7), mRNA. |
| **EBF1** | -1.70 | 0.00234 | 1879 | EBF1 | early B-cell factor 1 (EBF1), mRNA. |
| **NR2F1*** | -1.68 | 0.000493 | 7025 | NR2F1 | nuclear receptor subfamily 2, group F, member 1 (NR2F1), mRNA. |
| **C1QTNF5** | -1.67 | 0.000783 | 114902 | C1QTNF5 | C1q and tumor necrosis factor related protein 5 (C1QTNF5), mRNA. |
| **ID3** | -1.66 | 0.006719 | 3399 | ID3 | inhibitor of DNA binding 3, dominant negative helix-loop-helix protein (ID3), mRNA. |
| **NES** | -1.60 | 0.008058 | 10763 | NES | nestin (NES), mRNA. |
| **MAN1C1** | -1.46 | 0.000269 | 57134 | MAN1C1 | mannosidase, alpha, class 1C, member 1 (MAN1C1), mRNA. |
| **CYP1B1** | -1.45 | 0.009841 | 1545 | CYP1B1 | cytochrome P450, family 1, subfamily B, polypeptide 1 (CYP1B1), mRNA. |
| **PDK4** | -1.45 | 0.008942 | 5166 | PDK4 | pyruvate dehydrogenase kinase, isozyme 4 (PDK4), mRNA. |
| **PID1** | -1.43 | 0.001039 | 55022 | PID1 | phosphotyrosine interaction domain containing 1 (PID1), mRNA. |
| **LOC205251** | -1.43 | 0.002299 | 205251 | LOC205251 | PREDICTED: misc_RNA (LOC205251), miscRNA. |
| **GUCY1A3** | -1.43 | 0.0013 | 2982 | GUCY1A3 | guanylate cyclase 1, soluble, alpha 3 (GUCY1A3), mRNA. |
| **PDE7B** | -1.41 | 0.00369 | 27115 | PDE7B | phosphodiesterase 7B (PDE7B), mRNA. |
| **HSPB2** | -1.37 | 0.002628 | 3316 | HSPB2 | heat shock 27kDa protein 2 (HSPB2), mRNA. |
| **ITPR1** | -1.37 | 0.007249 | 3708 | ITPR1 | inositol 1,4,5-triphosphate receptor, type 1 (ITPR1), transcript variant 2, mRNA. |
| **CA11** | -1.37 | 0.008935 | 770 | CA11 | carbonic anhydrase XI (CA11), mRNA. |
| **VAMP5** | -1.34 | 0.001746 | 10791 | VAMP5 | vesicle-associated membrane protein 5 (myobrevin) (VAMP5), mRNA. |
| **GLI3** | -1.33 | 0.002368 | 2737 | GLI3 | GLI-Kruppel family member GLI3 (Greig cephalopolysyndactyly syndrome) (GLI3), mRNA. |
| **BIN1** | -1.32 | 0.006248 | 274 | BIN1 | bridging integrator 1 (BIN1), transcript variant 1, mRNA. |
| **C6ORF160** | -1.31 | 0.000967 | 387066 | C6orf160 | PREDICTED: chromosome 6 open reading frame 160, transcript variant 4 (C6orf160), mRNA. |
| **SNCG** | -1.31 | 0.004013 | 6623 | SNCG | synuclein, gamma (breast cancer-specific protein 1) (SNCG), mRNA. |
| **SNHG5** | -1.31 | 0.00101 | 387066 | C6orf160 | PREDICTED: chromosome 6 open reading frame 160, transcript variant 4 (C6orf160), mRNA. |
| **CXORF57** | -1.27 | 0.000113 | 55086 | CXorf57 | chromosome X open reading frame 57 (CXorf57), mRNA. |
| **GHR** | -1.24 | 0.000543 | 2690 | GHR | growth hormone receptor (GHR), mRNA. |
| **TCEAL3** | -1.17 | 0.009006 | 85012 | TCEAL3 | transcription elongation factor A (SII)-like 3 (TCEAL3), transcript variant 2, mRNA. |
| **LIX1L** | -1.17 | 0.000434 | 128077 | LIX1L | Lix1 homolog (mouse)-like (LIX1L), mRNA. |
| **IPO8** | -1.15 | 0.008381 | 10526 | IPO8 | importin 8 (IPO8), mRNA. |
| **CTSF** | -1.15 | 0.00529 | 8722 | CTSF | cathepsin F (CTSF), mRNA. |
| **ITPKB** | -1.11 | 0.009138 | 3707 | ITPKB | inositol 1,4,5-trisphosphate 3-kinase B (ITPKB), mRNA. |
| **FXYD1** | -1.11 | 0.006709 | 5348 | FXYD1 | FXYD domain containing ion transport regulator 1 (FXYD1), transcript variant a, mRNA. |
| **CHST3** | -1.10 | 0.001731 | 9469 | CHST3 | carbohydrate (chondroitin 6) sulfotransferase 3 (CHST3), mRNA. |
| **SLC9A9** | -1.05 | 0.00107 | 285195 | SLC9A9 | solute carrier family 9 (sodium/hydrogen exchanger), member 9 (SLC9A9), mRNA. |
| **AQP1** | -1.01 | 0.005075 | 358 | AQP1 | aquaporin 1 (Colton blood group) (AQP1), mRNA. |
