# Supplementary material for: Microarray Analysis Reveals Distinct Gene Expression Profiles Among Different Tumor Histology, Stage and Disease Outcomes in Endometrial Adenocarcinoma
Source: PLoS One. 2010 Nov 8;5(11):e15415. doi: 10.1371/journal.pone.0015415 (PMC2975707; doi:10.1371/journal.pone.0015415)
Supplement: Table S3 — The list of DEGs with at least two-fold change obtained from comparisons of late stage vs. early stage in EAC group. (DOC) [file pone.0015415.s003.doc]

**Table S3** The list of DEGs with at least two-fold change obtained from comparisons of late stage vs. early stage in EAC group.

| **Illumina ID** | **Log2 FC** | **P.Value** | **ENTREZ** | **SYMBOL** | **Description** |
| --- | --- | --- | --- | --- | --- |
| **TFF3*** | 2.90 | 0.002422 | 7033 | TFF3 | trefoil factor 3 (intestinal) (TFF3), mRNA. |
| **LOC652493** | 2.26 | 0.00086 | 652493 | LOC652493 | PREDICTED: similar to Ig kappa chain V-I region HK102 precursor (LOC652493), mRNA. |
| **LOC647450** | 1.88 | 0.001132 | 647450 | LOC647450 | PREDICTED: similar to Ig kappa chain V-I region HK101 precursor (LOC647450), mRNA. |
| **LOC649923** | 1.62 | 0.000107 | 649923 | LOC649923 | PREDICTED: similar to Ig gamma-2 chain C region (LOC649923), mRNA. |
| **LOC652694** | 1.60 | 0.002403 | 652694 | LOC652694 | PREDICTED: similar to Ig kappa chain V-I region HK102 precursor (LOC652694), mRNA. |
| **ELF5*** | 1.58 | 0.00176 | 2001 | ELF5 | E74-like factor 5 (ets domain transcription factor) (ELF5), transcript variant 2, mRNA. |
| **MGC29506** | 1.24 | 0.000118 | 51237 | MGC29506 | hypothetical protein MGC29506 (MGC29506), mRNA. |
| **FASN** | 1.22 | 0.001325 | 2194 | FASN | fatty acid synthase (FASN), mRNA. |
| **IGJ** | 1.18 | 0.001822 | 3512 | IGJ | immunoglobulin J polypeptide, linker protein for immunoglobulin alpha and mu polypeptides (IGJ), mRNA. |
| **WAS** | 1.14 | 0.007833 | 7454 | WAS | Wiskott-Aldrich syndrome (eczema-thrombocytopenia) (WAS), mRNA. |
| **CENPN** | 1.11 | 0.009505 | 55839 | CENPN | centromere protein N (CENPN), mRNA. |
| **CD8A** | 1.07 | 0.002934 | 925 | CD8A | CD8a molecule (CD8A), transcript variant 2, mRNA. |
| **LOC440731** | 1.06 | 0.007461 | 440731 | LOC440731 | PREDICTED: hypothetical LOC440731, transcript variant 2 (LOC440731), mRNA. |
| **ARMCX2** | 1.03 | 0.004356 | 9823 | ARMCX2 | armadillo repeat containing, X-linked 2 (ARMCX2), mRNA. |
| **CNTN1*** | 1.02 | 0.001517 | 1272 | CNTN1 | contactin 1 (CNTN1), transcript variant 1, mRNA. |
| **KIF14*** | 1.02 | 0.006701 | 9928 | KIF14 | kinesin family member 14 (KIF14), mRNA. |
| **FBXO5** | 1.01 | 0.009699 | 26271 | FBXO5 | F-box protein 5 (FBXO5), mRNA. |
| **ENPP3** | 1.00 | 0.00565 | 5169 | ENPP3 | ectonucleotide pyrophosphatase/phosphodiesterase 3 (ENPP3), mRNA. |
| **LMNB2** | 1.00 | 0.002267 | 84823 | LMNB2 | lamin B2 (LMNB2), mRNA. |
| **HOXD10*** | -1.25 | 0.009381 | 3236 | HOXD10 | homeobox D10 (HOXD10), mRNA. |
| **PYY** | -1.07 | 0.00987 | 5697 | PYY | peptide YY (PYY), mRNA. |
| **C9ORF58** | -1.06 | 0.006638 | 83543 | C9orf58 | chromosome 9 open reading frame 58 (C9orf58), transcript variant 1, mRNA. |
| **GALNTL1** | -1.04 | 0.006876 | 57452 | GALNTL1 | UDP-N-acetyl-alpha-D-galactosamine:polypeptide N-acetylgalactosaminyltransferase-like 1 (GALNTL1), mRNA. |
