# Supplementary material for: Microarray Analysis Reveals Distinct Gene Expression Profiles Among Different Tumor Histology, Stage and Disease Outcomes in Endometrial Adenocarcinoma
Source: PLoS One. 2010 Nov 8;5(11):e15415. doi: 10.1371/journal.pone.0015415 (PMC2975707; doi:10.1371/journal.pone.0015415)
Supplement: Table S4 — The list of DEGs with at least two-fold change obtained from comparisons of good prognosis vs. poor prognosis in USC group. (DOC) [file pone.0015415.s004.doc]

**Table S4** The list of DEGs with at least two-fold change obtained from comparisons of good prognosis vs. poor prognosis in USC group.

| **Illumina ID** | **Log2 FC** | **P.Value** | **ENTREZ** | **SYMBOL** | **Description** |
| --- | --- | --- | --- | --- | --- |
| **DLK1** | 4.62 | 4.48E-05 | 8788 | DLK1 | delta-like 1 homolog (Drosophila) (DLK1), mRNA. |
| **TNMD** | 3.04 | 0.00377 | 64102 | TNMD | tenomodulin (TNMD), mRNA. |
| **CPXM1** | 2.16 | 0.00401 | 56265 | CPXM1 | carboxypeptidase X (M14 family), member 1 (CPXM1), mRNA. |
| **CYTL1** | 1.87 | 0.003041 | 54360 | CYTL1 | cytokine-like 1 (CYTL1), mRNA. |
| **RHOBTB3*** | 1.58 | 0.009793 | 22836 | RHOBTB3 | Rho-related BTB domain containing 3 (RHOBTB3), mRNA. |
| **RNF24** | 1.55 | 0.000127 | 11237 | RNF24 | ring finger protein 24 (RNF24), mRNA. |
| **ZFHX4** | 1.47 | 0.00378 | 79776 | ZFHX4 | zinc finger homeobox 4 (ZFHX4), mRNA. |
| **LOC650200** | 1.46 | 0.001931 | 650200 | LOC650200 | PREDICTED: similar to Nuclear protein 1 (Protein p8) (Candidate of metastasis 1) (LOC650200), mRNA. |
| **C3ORF54** | 1.43 | 0.003701 | 389119 | C3orf54 | chromosome 3 open reading frame 54 (C3orf54), mRNA. |
| **SERPINH1** | 1.43 | 0.007804 | 871 | SERPINH1 | serpin peptidase inhibitor, clade H (heat shock protein 47), member 1, (collagen binding protein 1) (SERPINH1), mRNA. |
| **CEBPA*** | 1.35 | 0.000984 | 1050 | CEBPA | CCAAT/enhancer binding protein (C/EBP), alpha (CEBPA), mRNA. |
| **COL22A1** | 1.35 | 0.006032 | 169044 | COL22A1 | collagen, type XXII, alpha 1 (COL22A1), mRNA. |
| **OLFM2** | 1.29 | 0.006088 | 93145 | OLFM2 | olfactomedin 2 (OLFM2), mRNA. |
| **LOC400406** | 1.22 | 0.000291 | 400406 | LOC400406 | PREDICTED: similar to ADAM metallopeptidase with thrombospondin type 1 motif, 7 preproprotein (LOC400406), mRNA. |
| **C20ORF55** | 1.09 | 0.006762 | 83541 | C20orf55 | chromosome 20 open reading frame 55 (C20orf55), transcript variant 1, mRNA. |
| **IL27RA** | 1.09 | 0.001883 | 9466 | IL27RA | interleukin 27 receptor, alpha (IL27RA), mRNA. |
| **LOC642412** | 1.05 | 0.006077 | 642412 | LOC642412 | PREDICTED: similar to matrix-remodelling associated 8 (LOC642412), mRNA. |
| **RRBP1** | 1.05 | 0.002051 | 6238 | RRBP1 | ribosome binding protein 1 homolog 180kDa (dog) (RRBP1), transcript variant 1, mRNA. |
| **EIF3G** | 1.05 | 0.005934 | 8666 | EIF3G | eukaryotic translation initiation factor 3, subunit G (EIF3G), mRNA. |
| **FOSB*** | -2.24 | 0.008337 | 2354 | FOSB | FBJ murine osteosarcoma viral oncogene homolog B (FOSB), mRNA. |
| **REC8** | -1.95 | 0.008971 | 9985 | REC8 | REC8 homolog (yeast) (REC8), transcript variant 1, mRNA. |
| **RASSF7*** | -1.74 | 0.002621 | 8045 | RASSF7 | Ras association (RalGDS/AF-6) domain family (N-terminal) member 7 (RASSF7), mRNA. |
| **C11ORF9** | -1.67 | 0.008739 | 745 | C11orf9 | chromosome 11 open reading frame 9 (C11orf9), transcript variant 1, mRNA. |
| **PTPLA** | -1.62 | 0.00186 | 9200 | PTPLA | protein tyrosine phosphatase-like (proline instead of catalytic arginine), member A (PTPLA), mRNA. |
| **GJC2** | -1.41 | 0.007127 | 57165 | GJC2 | gap junction protein, gamma 2, 47kDa (GJC2), mRNA. |
| **FKBP11** | -1.40 | 0.006825 | 51303 | FKBP11 | FK506 binding protein 11, 19 kDa (FKBP11), mRNA. |
| **PDXK** | -1.34 | 0.000752 | 8566 | PDXK | pyridoxal (pyridoxine, vitamin B6) kinase (PDXK), mRNA. |
| **HS.568741** | -1.21 | 0.001468 | NA |  | cDNA FLJ26539 fis, clone KDN09310 |
| **PALM** | -1.20 | 0.007817 | 5064 | PALM | paralemmin (PALM), transcript variant 1, mRNA. |
| **HS.181245** | -1.19 | 0.004389 | NA |  | cDNA FLJ26539 fis, clone KDN09310 |
| **ATP5G1** | -1.12 | 0.000751 | 516 | ATP5G1 | ATP synthase, H+ transporting, mitochondrial F0 complex, subunit C1 (subunit 9) (ATP5G1), nuclear gene encoding mitochondrial protein,  transcript variant 2, mRNA. |
| **SCNN1D** | -1.12 | 0.002522 | 6339 | SCNN1D | sodium channel, nonvoltage-gated 1, delta (SCNN1D), transcript variant 2, mRNA. |
| **C21ORF2** | -1.12 | 0.007181 | 755 | C21orf2 | chromosome 21 open reading frame 2 (C21orf2), mRNA. |
| **C9ORF123** | -1.08 | 0.004211 | 90871 | C9orf123 | chromosome 9 open reading frame 123 (C9orf123), mRNA. |
| **HS.527657** | -1.06 | 0.004519 | NA |  | cDNA FLJ26539 fis, clone KDN09310 |
| **TMEM141** | -1.01 | 0.004218 | 85014 | TMEM141 | transmembrane protein 141 (TMEM141), mRNA. |
