# Supplementary material for: Microarray Analysis Reveals Distinct Gene Expression Profiles Among Different Tumor Histology, Stage and Disease Outcomes in Endometrial Adenocarcinoma
Source: PLoS One. 2010 Nov 8;5(11):e15415. doi: 10.1371/journal.pone.0015415 (PMC2975707; doi:10.1371/journal.pone.0015415)
Supplement: Table S5 — The list of DEGs with at least two-fold change obtained from comparisons of good prognosis vs. poor prognosis in EAC group. (DOC) [file pone.0015415.s005.doc]

**Table S5** The list of DEGs with at least two-fold change obtained from comparisons of good prognosis vs. poor prognosis in EAC group.

| **Illumina ID** | **Log2 FC** | **P.Value** | **ENTREZ** | **SYMBOL** | **Description** |
| --- | --- | --- | --- | --- | --- |
| **SLCO2A1** | 2.58 | 0.004573 | 6578 | SLCO2A1 | solute carrier organic anion transporter family, member 2A1 (SLCO2A1), mRNA. |
| **SLC47A1** | 2.42 | 0.002001 | 55244 | SLC47A1 | solute carrier family 47, member 1 (SLC47A1), mRNA. |
| **LEFTY2** | 2.39 | 0.007703 | 7044 | LEFTY2 | left-right determination factor 2 (LEFTY2), mRNA. |
| **FBLN1*** | 2.27 | 0.003646 | 2192 | FBLN1 | fibulin 1 (FBLN1), transcript variant D, mRNA. |
| **SLC16A9** | 1.85 | 0.005772 | 220963 | SLC16A9 | solute carrier family 16, member 9 (monocarboxylic acid transporter 9) (SLC16A9), mRNA. |
| **GJA4** | 1.83 | 0.005341 | 2701 | GJA4 | gap junction protein, alpha 4, 37kDa (GJA4), mRNA. |
| **EPDR1** | 1.82 | 0.001022 | 54749 | EPDR1 | ependymin related protein 1 (zebrafish) (EPDR1), mRNA. |
| **C9ORF61** | 1.65 | 0.004424 | 9413 | C9orf61 | chromosome 9 open reading frame 61 (C9orf61), mRNA. |
| **PPP2R2C** | 1.53 | 0.001814 | 5522 | PPP2R2C | protein phosphatase 2 (formerly 2A), regulatory subunit B, gamma isoform (PPP2R2C), transcript variant 1, mRNA. |
| **NT5E** | 1.51 | 0.002794 | 4907 | NT5E | 5'-nucleotidase, ecto (CD73) (NT5E), mRNA. |
| **GREM2** | 1.39 | 0.006266 | 64388 | GREM2 | gremlin 2, cysteine knot superfamily, homolog (Xenopus laevis) (GREM2), mRNA. |
| **SETBP1** | 1.33 | 0.004654 | 26040 | SETBP1 | SET binding protein 1 (SETBP1), mRNA. |
| **PAGE4** | 1.30 | 0.000465 | 9506 | PAGE4 | P antigen family, member 4 (prostate associated) (PAGE4), mRNA. |
| **RGL1** | 1.22 | 0.007799 | 23179 | RGL1 | ral guanine nucleotide dissociation stimulator-like 1 (RGL1), mRNA. |
| **C22ORF36** | 1.18 | 0.008538 | 388886 | C22orf36 | chromosome 22 open reading frame 36 (C22orf36), mRNA. |
| **APLNR*** | 1.17 | 0.006882 | 187 | APLNR | apelin receptor (APLNR), mRNA. |
| **PGR** | 1.13 | 0.001463 | 5241 | PGR | progesterone receptor (PGR), mRNA. |
| **ANAPC4** | 1.11 | 0.005881 | 29945 | ANAPC4 | anaphase promoting complex subunit 4 (ANAPC4), mRNA. |
| **HSPA5** | 1.03 | 0.005589 | 3309 | HSPA5 | heat shock 70kDa protein 5 (glucose-regulated protein, 78kDa) (HSPA5), mRNA. |
| **KIAA1826** | 1.03 | 0.001635 | 84437 | KIAA1826 | KIAA1826 (KIAA1826), mRNA. |
| **MYOM2** | 1.02 | 0.002056 | 9172 | MYOM2 | myomesin (M-protein) 2, 165kDa (MYOM2), mRNA. |
| **HS.379253** | 1.01 | 0.00416 | NA |  | cDNA FLJ26539 fis, clone KDN09310 |
| **IL20RA** | 1.00 | 0.007389 | 53832 | IL20RA | interleukin 20 receptor, alpha (IL20RA), mRNA. |
| **HOXD1** | -1.86 | 0.007317 | 3231 | HOXD1 | homeobox D1 (HOXD1), mRNA. |
| **SPRYD5** | -1.80 | 0.001843 | 84767 | SPRYD5 | SPRY domain containing 5 (SPRYD5), mRNA. |
| **ARID3A** | -1.67 | 0.000925 | 1820 | ARID3A | AT rich interactive domain 3A (BRIGHT-like) (ARID3A), mRNA. |
| **ASCL2** | -1.61 | 0.007613 | 430 | ASCL2 | achaete-scute complex homolog 2 (Drosophila) (ASCL2), mRNA. |
| **FABP5** | -1.54 | 0.008728 | 2171 | FABP5 | fatty acid binding protein 5 (psoriasis-associated) (FABP5), mRNA. |
| **NMU** | -1.41 | 0.007164 | 10874 | NMU | neuromedin U (NMU), mRNA. |
| **PFKFB4** | -1.39 | 0.002707 | 5210 | PFKFB4 | 6-phosphofructo-2-kinase/fructose-2,6-biphosphatase 4 (PFKFB4), mRNA. |
| **KCNJ16** | -1.25 | 0.000409 | 3773 | KCNJ16 | potassium inwardly-rectifying channel, subfamily J, member 16 (KCNJ16), transcript variant 3, mRNA. |
| **LOC731049** | -1.20 | 0.006189 | 731049 | LOC731049 | PREDICTED: similar to Ubiquitin-conjugating enzyme E2S (Ubiquitin-conjugating enzyme E2-24 kDa) (Ubiquitin-protein ligase)  (Ubiquitin carrier protein) (E2-EPF5) (LOC731049), mRNA. |
| **SLC2A5** | -1.18 | 0.006252 | 6518 | SLC2A5 | solute carrier family 2 (facilitated glucose/fructose transporter), member 5 (SLC2A5), mRNA. |
| **FST*** | -1.17 | 0.007778 | 10468 | FST | follistatin (FST), transcript variant FST317, mRNA. |
| **GPI** | -1.12 | 0.00025 | 2821 | GPI | glucose phosphate isomerase (GPI), mRNA. |
| **TNFRSF11B** | -1.12 | 0.000857 | 4982 | TNFRSF11B | tumor necrosis factor receptor superfamily, member 11b (TNFRSF11B), mRNA. |
| **MPP6** | -1.10 | 0.009617 | 51678 | MPP6 | membrane protein, palmitoylated 6 (MAGUK p55 subfamily member 6) (MPP6), mRNA. |
| **LAP3** | -1.09 | 0.009202 | 51056 | LAP3 | leucine aminopeptidase 3 (LAP3), mRNA. |
| ***LMO4**** | -1.00 | 0.008869 | 8543 | LMO4 | LIM domain only 4 (LMO4), mRNA. |
